# Medical Items and News of Interest to the Physician

**Published:** 1880-10

**Authors:** 


### For the Use of Physicians.

CULLED FROM THE STANDARD MEDICAL JOURNALS OF THE WORLD.

**Chronic Eczema.**

Calomel 15 to 25 grains.
Tannin, 40 "
Glycerite of starch, 1 ounce.

**Coca as a Cure for Opium-Habit.**

Dr. J. G. Cove reports a cure of morphia-habit following the use of coca which was taken *ad libitum*.–*Louisville Med. News.*

**Rheumatism,**

Olive oil, 2 lbs.
Powdered chillies, ½ lb.

Boil for six hours and add 6 oz. melted spermaceti. Scent with oil of lavender. To be applied as a poultice, q. s.—W. H. BROWN, in *Pharm. Jour.*

**Burns.**—*Ungentum Iodoformi Compositum.*

℞
Iodoformi, 80 grains.
Extr. conii, 40 "
Acidi carbol, 10 min.
Ung. aquæ rosæ, 1 ℥

M. To be applied twice daily, with picked lint, upon the burnt parts.—*Pharm. Centralh.*

**Vomiting of Pregnancy.**

Dr. Hickman claims that for this purpose Oxalate of Cerium should be given much more freely than is customary—ten grains as often as necessary, taking care to give the first dose half an hour before the patient rises from bed. He has not seen goods results follow a combination of it with bismuth.—*Med. and Surg. Rep.*

**Copaiba-Mixture.**

The following formula is recommended as a good method for administering this disagreeable remedy. As it is apt to leave a caustic taste in the mouth, it is advisable to rinse the mouth out with some mild diluent, such as barley water.

℞

Copaibæ, ʒ ij.

Acaciæ pulv., ʒ ij.

Sacchari pulv., ʒ iij.

Liq. potassæ, fl. ʒ jss.

Spir. myristicæ, fl. ʒ j.

Aquæ cinnamomi, q. s. ad fl. ℥ vj.

*Dose:* A tablespoonful twice daily.

**Diphtheria.**

Lactic acid, 10 parts,
Water. 30 parts, M.

Dip a pencil into the liquid, and paint the false membranes with it every 15 minutes. At the same time a mixture of chlorate of potassium and of chloride of iron is administered internally. Every two or three hours some nourishment is given, and in the sick chamber an abundant supply of moisture is to be maintained.—D. KINGSFORD in *Petit Mon. de la Med.*

**Antidote for Strychnia, Etc.**

According to Prof. Theodor Husemann, chloral is a powerful antidote for strychnia, brucia and thebaia, having preserved life even in cases where quantities amounting to six times the fatal dose of these poisons had been taken. The quantity of chloral to be administered at a time should not be less than 2 gm. (31 grains) and not over 3 gm. (46 grs.)

**Oleate of Lead.**

Dr. James Sawyer, thinking to try the effects of oleate of lead as a remedy for eczema, had a preparation made by Messrs. Southall, of Birmingham, as follows : An oleate of lead is prepared by heating oleic acid with oxide of lead [proportions not stated—ED.], and 24 parts of this are mixed with 14 parts of inodorous paraffin oil. He has used it for several months and finds it to be an efficient remedy.—*Br. Med. Jour.*

**Tetanus.**

Dr. John C. Lucas, in the *Medical Times and Gazette*, strongly advocates the treatment of tetanus by smoking Indian hemp. The leaves of the cannabis indica are mixed with three or four times their quantity of ordinary tobacco. Directly there are indications of a spasm coming on, the fumes are inhaled until the attack ceases. The patient is then left quiet, but carefully watched, so that the pipe may be instantly handed to him on any appearance of the spasm returning. In this way the patient is kept continuously under the influence of hemp, day and night, nourishment being carefully administered at the same time. The advantages claimed for this mode of treatment are these : 1. The spasms are cut short. 2. They reappear gradually, at longer and longer intervals. 3. They gradually become not only less frequent, but less severe. 4. This saves the patient's vital powers. Mr. Khasligir, of India, has also treated five cases of traumatic tetanus, all recovering, by this method.

**Mistura Balsami Copaivæ.**—*Copaiba Mixture.*

| | |
|---|---|
| Copaiba, | 8 parts. |
| Alcohol, 70 per cent., | 8 " |
| Syrup of orange flowers, | 8 " |
| Peppermint water, | 8 " |
| Orange flower water, | 2 " |
| Spirit of nitrous ether, | 1 part. |

—*Russian Pharm. of* 1880.

**Signs of Death.**

M. Beyraud, of Libourne, in a commnication to the Academy of Sciences in Paris, writes that real death may be recognized in a practical manner by means of the application of the cautery to the supposed corpse. If the eschar do not show itself, the subject is dead; if it be yellow and transparent, the subject is dead; if it be black or of a reddish-brown, the subject is living.

**Vaginitis.**

Dr. Gallord introduces small suppositories at first, and gradually enlarging—each being made of iodoform and belladonna. At first the iodoform, thirty grains, is mixed with cocoa butter, thirty grains, and fresh lard seven times as much. If there be pain without much mucous inflammation, he adds the extract belladonna with lard, in similar weights. After some little time the cure is effected.

**Duboisia and Atropia.**

Dr. Sidney Ringer, in the Practitioner, says: "Dubosia possesses the same properties as atropia, but is far more powerful than atropia. Mr. Tweedy found this to be the case in regard to the local application to the eye. But while duboisia is far more powerful than atropia on man, the reverse is the case in respect to frogs. Atropia paralyzes far more powerfully the motor nervous system, the heart and respiration, in frogs than duboisia."

**Internal Use of Tar.**

In a recent number of the Berliner Klin. Wochenschrift, Prof. Reclam, of Leipzic, reports some therapeutic experiments with tar. He used it in pills or capsules, and says that tar water is not so efficient. One curious and constant effect noted was that the urine of a patient taking tar does not decompose for five or six days, instead of in twenty-four hours as usual. The general indication for tar, he says, is a chronic catarrhal inflammation of the mucous passages of the respiratory or urinary tract as bronchitis, vesical catarrh, gleet, etc.

**Caustic Crayons.**

Five parts of silver, with one part nitrate of of nitrate of lead, being melted together, can be cast into crayons which are not easily broken, and which may be sharpened to a point, like a lead-pencil. This is a suggestion of Dr. Sawostiki, of St. Petersburg.—*Boston Med. and Surg. Jour.*

**Chloroform Gargle.**

Alcohol, 100 parts,
Chloroform, 5 to 10 parts,
Oil of peppermint, 5 to 10 parts.

This is said by Dr. Schroffer to unite the properties of a disinfectent, roborant and anodyne, for various forms of dental narcosis The same author also found chloroform to be the most prompt agent for arresting bleeding after the extraction of a tooth, by washing out the alveolus with it.—*Ph. Zeit. f. Russl.*

**Administration of Castor Oil.**

The following method of administering castor oil is recommended by M. Potain, and described in a recent number of Le Practicien: An orange is cut in halves, and after removing the pips, the juice of one half is pressed out into a teacup. The oii is poured carefully on the top of this juice, and on this again the juice of the other half of the orange is squeezed out. The oil remains between the two layers of juice in the shape of a meniscus, and may be swallowed without any unpleasant taste.

**Chian Turpentine,**

The introduction of new remedial agents will necessarily create fresh difficulties for the dispenser, and in doing so will tax his resources to the utmost, and the same wil equally apply to old remedies exhibited under a new aspect. These instances are not by any means rare. There is one striking illustration in the resuscitation of that very old and long disused drug, chian turpentine. The pharmacy of this substance either alone or combined with sulphur, has brought forth numerous letters from advocates of several modes of combining chian turpentine to form an emulsion, presentable, and at the same time palatable, in fact, a suitable method of administering this very scarce, and consequently very valuable drug. Happy must that pharmacist feel, who, with a veneration for old pots and their contents, has preserved his genuine chian turpentine for the benefit of suffering humanity and a considerable advance on its normal cost.
—*London (Eng.) Phar. Jour. and Tran.*

**Diabetes.**

Dr. H. Brubaker reports in *Clinical Record* a case of diabetes mellitus in a patient aged 70. The patient had been suffering for several years and was much emanciated. He had a fondness for maple sugar, and was finally told to eat it *ad libitum*, no restrictions being placed on this diet. From this time the sugar in his urine began to grow less, and finally entirely disappeared, as did the other diabetec symptoms. The patient died, however, from other causes.

---

**Chronic Laryngitis.**

Oil of Eucalyptus, 3 to 5 gm.=45 to 80 grs.
Alcohol, 75 " = 3 fl. oz.
Distilled water, 170 " = 5½ fl. oz.

Dissolve the oil in the alcohol, and gradually add the water. This liquid is introduced into a spray-apparatus, and applied four times daily, for 10 or 15 minutes at a time. The vapors produce a copious expectoration. It is beneficial in bronchitis and in chronic laryngitis; also in catarrh of the nasal fossæ, and of the pharynx.—M. Mosler in *Petit Mon. de la Med.*

---

**Cedron as Substitute for Quinine.**

Admiral Lapellin draws attention to the bean which is used by the inhabitants of Central America in the treatment of the cold fever, and said to be a good substitute for quinine. Dr. Coignard, who obtained the remedy in Puerto Arenas, Costa Rica, obtained favorable results with it, and Drs. St. Pere and Quesnel found it even more powerful than sulphate of quinine. The bean is cut into bits as large as a pea, several of which are given in the interval between the paroxysms. This almond or bean is obtained from the simaruba ferruginea.—*Med. Chir. Rundschau*, Nov., 1879.—*The Med. Record.*

---

**A New Anthelmintic.**

Dr. Lemos, in *Medicinische Neuigkeiten*, states that the ocinum basilicum, a plant known in Buenos Ayres under the name "albochaca," has an action of such a nature that the worms in every stage of development rapidly leave their location after the juice reaches them. Its use is so much the more to be recommended, since, if no worms should be present no injurious effect results from the plant, but a laxative and disinfectant action is the only result. Fifty grammes of the juice are given, followed in two hours by a dose of castor oil. A free discharge of the worms may be expected.—*Phila. Med. and Surg. Reporter.*

**Gonorrhea.**

Prof. J. G. Hyndman, of Cincinnati, has had remarkable success in the treatment of five cases of gonorrhea, from injecting once or twice a day a solution of boracic acid in water, 5 to 18 grains to the ounce. Two or three days was sufficient in some instances to effect a cure. He advises the five grain solution to begin with. In a female with profuse discharge, the ten grain solution cured in three days. We are rather surprised at a statement made by Dr. Hyndman that this disease is known to be more difficult to cure in persons who have had previous attacks.—*Pacific Medical Journal.*

---

**Chronic Eczema.**

Dr. C. T. Poe of Grand Island, Neb., writes to the *Ther. Gaz.*, as follows:

"I have given berberis aquifolium a fair trial as an alterative in the treatment of a severe case of chronic eczema. Patient eighteen months old, disease one year's standing, eruption covering whole surface of body. After giving all the usual remedies a fair trial, as a last resort, I obtained two ounces of berberis aquifolium. Ordered five drops four times in twenty-four hours. Three-fourths of the quantity is taken, and the hideous little monster is now a lovely child, well, bright and happy."

---

**Oil of Tansy.**

Dr. G. Jewett, *Boston Medical and Surgical Journal*, reports eight casas of poisoning with this drug. Case 1. Fifteen drops at 11 A. M., a teaspoonful at 2 P. M.; convulsions, shock, general cyanosis; recovery. Case 2. Teaspoonful to promote catamenia; convulsions. and death in one hour and a half. Case 3, Unknown quantity to cause abortion; convulsions; death in three hours and a quarter; no abortion. Case 4. Teaspoonful to cause abortion; coma; recovery; no abortion. Case 5. Four drachms; spasms and death. Case 6. To cause abortion; rapid death; no abortion. Case 7. Decoction of tanzy leaves to produce abortion; paralysis; coma; death in twenty-four hours without abortion. Case 8. Infusion of leaves daily for a week; also for vaginal injection; abortion, metritis, peritonitis; recovery after three months.

As druggists are often asked for oil of tansy under various pretences, we believe the above table will be useful in reminding them of the dangers attending the sale of tanzy and its preparations.

**Relation of Diarrhœa to Temperature.**

The unusually cold and damp summer experienced in Great Britain last year has resulted in an extraordinary reduction in the number of deaths from diarrhœa. We see it stated that at Birmingham, where the mean temperature was 2.6° lower than that for the corresponding period of 1878, there were only 81 deaths from diarrhœa last July, August, and September, against 534 in those months in 1878.

---

**Citrate of Caffeine.**

Dr. Leech lately read before the Manchester Medical Society a paper on the therapeutic uses of citrate of caffeine. His remarks were chiefly devoted to its diuretic action, and he showed by tabulating the quantity of urine passed under the influence of different drugs, and apart form the influence of drugs, that caffeine has a powerful effect in increasing the action of the kidneys in some pathological conditions. He found it most useful in dropsy dependent on heart-disease, but in some obscure cases of ascites apparently due to peritoneal changes it had seemed to be of much service.

---

**Sick Headache.**

Dr. J. E. Stinson, of Montague, Texas, recommends the following mixture in the Peoria *Medical Monthly* for July, 1880:

℞
Tinct. opii.
Fl. ext. colocynth, aa ʒ ss.
"    valerianæ.
Spt. ether. comp., aa ℥ j.

M. Sig.—Give a teaspoonful every three hours until relieved.

After the attack has passed away he places his patient on the following nerve tonic :

℞

Fl. ext. colocynth, ʒ j.

"    nucis vom., ʒ ij.

"    cinchonæ, ℥ ij.

Spt. frumenti, ℥ iv.

M. Sig.—Teaspoonful five times daily, to be discontinued during the attacks.

---

**Administering Kooso.**

Of all the remedies for tape worm, none is more certain and efficient than kooso, and many efforts have been made to bring it into such pharmaceutical shape that, while its properties as a tænicide remain unimpaired, it might be administered without repugance. Dr. Corre, some years ago proposed the following method, which has been successfully used in many cases: One half ounce of freshly powdered kooso is treated with one ounce of hot castor oil, and afterwards with two ounces of boiling water by displacement ; express, and by means of the yolk of an egg combine the two percolates into an emulsion, and add forty drops of sulphuric ether, flavoring with some aromatic oil. This is to be taken in one dose early in the morning, after a previous fast of about eighteen hours. The worm is usually expelled dead after six or eight hours.—*Medical Press and Circular.*

---

**Local Anæsthesia with Bromide of Ethyl.**

M. Terrillon stated, before the Paris Surgical Society, that he had employed the bromide of ethyl about a dozen times in operations with the thermo-cautery. In a minute or two a white patch indicating cutaneous anæsthesia is produced, and on the pulverization being continued, insensibility of the tissues is produced to the depth of two centimetres. The production of the white patch is not essential, as anæsthesia may exist when it is absent. The results have proved very satisfactory, but in two cases M. Terrillon did not succeed, owing, as he believes, to the pulverizators which he employed having too small a jet.

---

**Dyspnœa.**

Quebracho appears to be, from the reports of Penzoldt and others, a very efficient palliative of all forms of dyspnœa. My experience of its efficacy refers only to a limited number of cases, in which the dyspnœa was associated with emphysema of the lungs, atheroma of the arteries, and degeneration of the cardiac muscles. In all these cases a teaspoonful of the liquid extract has afforded *immediate relief.* In three minutes after the administration of the drug, the pulse became somewhat fuller, but not increased in frequency ; the patients felt their breathing easier ; the face was flushed, and a gentle perspiration appeared on the forehead. There were slight drowsiness and inclination to sleep. These symptoms, however, soon subsided, while the breathing continued to be much improved. One patient, suffering from cardiac dropsy, stated that he had passed more urine after taking three doses of the medicine than he had done before. I had, however, no opportunity of verifying this statement.

From what I have seen of the action of the drug, I am confident that it is deserving of a fair trial.—*I. B. Berkart, M. D., in British Medical Journal.*

**To Facilitate the Exhibition of Iron.**

Professor T. Grainger Stewart, in *Practitioner*, draws attention to a certain condition which often arises in cardiac affections (particularly aortic disease), demanding for its treatment large doses of iron, and states that in some cases both belonging to the above groups and of other kinds, the reception of iron by the system is greatly facilitated if chloride of ammonium be administered along with it. The form of iron he finds best is the tincture of the perchloride; to this he adds chloride of ammonium, in doses of half a grain to each minim. He maintains that in cases in which iron, if given alone, causes dyspepsia, the latter is relieved by the combination described, and the patient is enabled to bear large doses of iron for a considerable time.

---

**Chest Diseases.**

Dr. Moubre, writing to the *Gazette de Hopitaux*, gives his experience of petroleum capsules in simple and chronic bronchitis. This balsamic had been brought before the Thereapeutic Society by Dr. Blache a year ago, at the instigation of a Paris chemist, who named it Gabian oil, in order to prevent public prejudice. Each capsule contains twenty-five centigrammes of pure petroleum, the ordinary oil not being used, as it has to be distilled in contact with sulphuric acid to render it fit for lighting purposes. At the Hospital Beaujon, where these capsules have been freely ordered for chronic bronchitis, a rapid diminution of the secretion and fits of coughing was observed. In tuberculosis this medicine gave encouraging results—*Philadelphia Medical and Surgical Reporter.*

---

**Fits of Sneezing.**

During the recent rapid changes of temperature I caught a severe cold in my head, accompanied by almost incessant sneezing. My unfortunate nose gave me no rest. The slightest impact of cold air, or passing from the outside air into a warm room, equally brought on a fit of sneezing. In vain I snuffed camphor and pulsatilla ; the light catarrh still triumphed over me. At length I resolved to see what the maintainance of an uniform temperature would do towards diminishing the irritability of my Schneiderian membrane, and accordingly I plugged my nostrils with cotton wool. The effect was instantaneous, I sneezed no more. Again and again I tested the efficacy of this simple remedy, always with the same result ; however near I was to a sneeze, the introduction of the pledgets stopped it *sur le champ.* Nor was there any inconvenience from their presence, making them sufficiently firm not to tickle, and yet leaving them sufficiently loose to easily breathe through. This is really worth knowing ; for an incessant sneezing is among the greatest of smaller ills ; and it seems only a rational conclusion to hope that, in this simple plan, we may have the most efficient remedy against one of the most distressing symptoms of hay-fever.—*S. Messenger Bradley, M. D., in British Med. Jour.*

---

**Hay Fever.**

Dr. R. H. Weber, of Philadelphia, communicates to the *American Journal of Pharmacy* a copy of the prescription which he has uniformly found useful in the complaint mentioned. Dr. Weber regards the iodide of potassium as the active agent, but the best results have always been obtained when combined with bicarbonate of potassium and hyoscyamus. The formula is as follows :

℞

Extracti hyoscyami, gr. xij.

Potassii iodidi, ʒ j.

Potassii bicarbonatis, ʒ ij.

Extr. glycyrrhizæ depurati, ʒ iv.

Aquæ anisi, ℥ ivss.

M. S.—A dessertspoonful every four hours, day and night, until relieved. The medicine is to be continued for at least a week, in doses of a dessertspoonful four times daily.

---

**Prurigo.**

From the observation of the fact that sufferers from prurigo feel relief when the secretion of the sudoriparous glands is active. O. Simon has been led to try the preparations of pilocarpine and of jaborandi itself in this distressing condition. A very numerous series of trials have persuaded him of the beneficial action of this means of treatment. In adults he uses a subcutaneous injection of pilocarpine, or prescribes a syrup of jaborandi. The patients, soon after the administration of the medicine, are enveloped in blankets for two or three hours. In patients suffering from psoriasis the perspiration is very scanty, while in pruriginous patients it is very abundant. The effects of this mode of treatment were, abatement of the accustomed sense of pruritus, softening of the skin, and diminished tendency to relapse. In general the case did not last longer than a fortnight, and in very severe cases three weeks.

**Nitrate of Amyl.**

Dr. Kerr gave five minims of nitrate of amyl by inhalation in a grave case of uterine hæmorrhage. The flow ceased at once and permanently, and the patient was restored from a state of collapse. Dr. Wm. F. Jenks, of Philadelphia, administered one-drop doses of the nitrate in puerperal eclampsia, and found it almost instantly arrested the paroxysms. He gave it at the approach of a convulsion. Contrary to Kerr, however, he found that its use was followed by extreme hæmorrhage and a general relaxation of a previously contracted uterus. Nevertheless, we believe if the uterus be protected by ergot and ice, that the nitrate of amyl would prove a most desirable and efficacious weapon against eclampsia.—*British Medical Journal.*

**Ulcers of the Cervix.**

Dr. Mendelsshon refers to thirty-seven cases in which the old treatment failed, and in which he had recourse to the following :

℞
Pure creosote, 30 grs.
Glycerine, 750 grs.
Alcohol, 375 grs.

M. He touched the ulcers three times a day with this mixture. Of twenty-eight cases, twenty-six had good results in two weeks. Of seven cases of gangrenous and granulating ulcers he had one complete cure, and most of the others improved. In two cancerous cases no improvement in forty days, and he began with iodoform. These results led him to believe the efficacy of creosote in non-specific ulcers. This drug, the author holds, owes its curative power over superficial ulcers to its astringent and antiseptic action.—*Ther. Gazette.*

**Aspidium Marginale in Tapeworm.**

The oleo-resin of this species of male fern was used successfully by Dr. Cressler, in 1878, as a tænicide. Prof. Maisch; at a recent meeting of the Pharmaceutical Society of this city, stated that he also had found it valuable. The oleo-resin, diluted with an equal bulk of alcohol, had been agitated with about fifteen or twenty times it weight of sugar, and afterwards with sufficient water to form a syrup. Given in this manner, in divided doses, like oleo-resin of male fern, it was well borne by the stomach. The same method had subsequently been tried by Mr. Kennedy with equal success, as reported by him to the Pennsylvania Pharmaceutical Association, at its last meeting. The only points to be observed were that the rhizome should be sound and free from any brown or decayed portions.—*Phila. Medical and Surgical Reporter.*

**Bladder aud Kidney Ailments.**

The following formula will give the most satisfactory results :

℞
Damianæ tinct.,
Hydrastis can. tinct., āā ℥ ij
Nucis vomicæ tinct., ʒ ij.
Sodæ hypophosph.,
Potassæ hypophosph., āā ʒ ij,
Syrupi aurantii cort.
Cardamoni comp. tinct., āā ℥ ss.
Aquae dest. q. s., ad. ℥ viij.

M. Sig. Dessert-spoonful after meals and at bedtime.

Although slower and more silent than buchu, uva ursi, and other positive diuretics, the true damiana, persistently administered, will meet every indication fulfilled by these drugs, and is and must prove their superior in all inflammatory and mucous membrane diseases of the genito-urinary apparatus. Damiana in combination with tincture cimicifuga is an elegant remedy in painful diseases of the muscular coat of the bladder, and in rheumatic or neurotic diseases involving the kidneys.

℞
Damianæ tinct., ℥ jss.
Cimicifugæ rac. tinct., ℥ ss.

M. Sig. Teaspoonful every four hours.

In painful hæmorrhagic diseases of the kidneys and bladder I have found nothing superior to—

℞
Damianæ tinct., ℥ ij.
Rhus glab. tinct., ℥ ij.
Morphiæ sulph. grs. iij.

M. Sig. Teaspoonful once in four hours.

In enlarged prostrate and acute and sub-acute inflammations of that gland, damiana in combination with phytolacca dec. and potassium bromide, will be found very valuable :

℞
Damianæ tinct., ℥ iij.
Phytolacca dec. tinct., ℥ j.
Potassii bromidi, ℥ ss.
Syrupi zingiberis, ℥ ss.
Aquæ dest. q. s., ad. ℥ vj.

M. Sig. Dessert-spoonful every four hours until patient is fully relieved ; afterward about once in six or eight hours until cured.

My conclusions are the result of two years close experimental observation.

**The Opium Habit.**

Since the publication in these columns of Professor Palmer's article on coca as an antidote to opium eating, the demand all over the country for the coca has been so great as to put the drug-houses to their best efforts to fill orders. Professor Palmer is daily in receipt of letters asking how the remedy is to be used. He asks us to publish the following: "Coca is to be used as a substitute for the opium. It is therefore to be taken as freely as the cravings of the system for opium may demand—tablespoonful doses of the fluid extract several times a day, more or less, as needed. The 'break-off' is to be made at once and for all, and coca is the staff upon which the sufferer is to throw his whole weight." He also asks that patients and physicians will send reports of results to him or to the editor of the *News*. He suggests that it is best that the drug should be given under the supervision of the family physician, so that any collateral contingencies may be met and counteracted.—*Louisville Medical News.*

---

**Diabetis Insipidus.**

In the May 8th, No. of the British Medical Journal, Dr. William Murrell recalls the report of a case of diabetes insipidus in the December 25th, 1875, No. of the same journal. This case was successfully treated with ergot after jaborandi and other remedies had been tried to no purpose. Half a drachm of the liquid extract was given every three hours, and the urine was reduced in twenty-four days from twenty pints to a pint and a half, the specific gravity being increased from 1002 to 1017. The excessive thirst and other distressing symptoms from which the patient suffered were also removed. A few days previous to his second reference to the case, Dr. Murrell accidentally met his quondam patient, who informed him he had not had a days illness since he had left the hospital, some four and a half years ago. His urine is normal in quantity and he does not suffer from thirst; and he is strong and well in every way, and able to do a good day's work. The ergot cured him completely.

In the May 22nd No. of the same journal, Dr. Samuel J. Noake corroborates Dr. Murrell's statement of the value of ergot in diabetes insipidus by the report of two cases which he treated with Dr. Canney, of Bishop Aukland, some twenty years ago. After the patient had been under his care for some time, he ordered the following: Ergot, one ounce, bruised, in a pint of cold water, and boiled to half a pint, strained and put into sixteen half ounce bottles, with directions for one to be taken every three hours. The specific gravity of the urine gradually increased under this treatment to 1021.

---

**Catarrh.**

Dr. Hermann Hager states that, being extremely subject to bronchial and pulmonary catarrh, in the spring and fall of the year, owing to sudden changes of weather, he has in the course of time, after trying various remedies, come to the conclusion that *quinine*, or another cinchona alkaloid, either had the power of entirely preventing the attack, or at least greatly to shorten it. He now prefers to take the remedy in pills, and he gives the following formula:

PILULÆ ANTIPHLOGISTICÆ, HAGER.

(Hager's Catarrh-Pills).

℞

| | | | |
|---|---|---|---|
| Quinida sulphate, | 5.0 gm. | = 75 grs. | |
| Cinchonidia sulph. | 5.0 " | = 75 " | |
| Tragacanth, pd., | 7.0 " | =108 " | |
| Althæ root, pd., | 3.0 " | = 45 " | |
| Gentian root, pd., | 3.0 " | = 45 " | |
| Red Saunders, | 1.0 " | = 15 " | |
| Glycerin, | 7.5 " | =115 " | |
| Hydrochloric acid, | 7.5 " | =115 " | |

Make 200 pills. Each should contain 0.05 gm. (= $\frac{7}{8}$ grain) of alkaloid.

On feeling the least discomfort, particularly after exposure, about 5 pills should be taken, and, if no relief is felt after an hour, 5 or 6 more. In more severe attacks the doses of 5 pills each may be repeated every half-hour until relief is felt. If the attaok supervenes at night, the repetition of a few doses generally insures a quiet night, undisturbed by cough.—From *Pharm. Centralh.*

---

**Skin Diseases.**

Dr. R. Farquharson, in a paper read before a section of the British Medical Association, speaking of the methods of administering arsenic, says that he prefers to give the remedy in decided doses, rather than begin with small doses, and increase it cautiously, believing that, like quinine and iodide of potassium, arsenic is more likely to produce well marked physiological effects when given in fairly large quantities than in small. As a rule, he begins with ten minims of liquor arsenicalis and pushes it boldly up to fifteen or twenty mimims. He seldom has much trouble from the patient's inability to continue the use of the drug.

He does not believe it to be necessary that the physiological effects of the drug should be produced, but that, on the contrary, it is apt to aggravate the disease. Abdominal tenderness and diarrhœa are less apt to occur when the remedy disagrees than sudden vomiting following each dose, and this is an effectual barrier to its use.

He does not consider that arsenic so much renders the patient susceptible to cold as that in relieving the skin affection it leads to a sort of metastasis to the mucous membrane of the air passages.

He prefers the liquor arsenicalis (Fowler's solution) to liq. sodii arsenitis. The dose should always be taken after or during a meal, and it is desirable to examine the urine occasionally during the use of the remedy, in order to be assured that the kidneys are working properly.—*Br. Med. Jour.*

---

**The Therapeutic Value of Pulsatilla.**

Pulsatilla is rapidly growing in favor with many practitioners. Though a very old remedy, having been known to Dioscorides and Pliny, it fell into disuse, if not into disrepute, and was not reinstated till about the beginning of the present century. I have used pulsatilla mainly in simple dysmenorrhœa, and here it has proved of decided utility. Its scope is, however doubtless much wider. A very prominent lawyer of this city told me, not long since, that after trying the bromides, the valerianates and other remedies of repute for the headaches caused by excessive mental application, he found no relief until he made use of the tincture of pulsatilla. He is now never without it, and uses no other medicine for the cure of his headaches, which I know to be very severe. No such powers are attributed to it in the books to which I have access. This is an exceptional case, it may be, but it is a valid one. The tincture of pulsatilla should be made from the fresh plant, and given with caution. The dose is from three to ten drops.—*J. J. Tucker, M. D., in Chicago Medical Gazette.*

---

**Asthma.**

Dr. A. E. Remington of Bullo, New Zeland, in a communication to the *Therapeutic Gazette*, recommends *grindelia robusta.* He writes :

"Since receiving the fluid extract grindelia I have used it for three persons suffering from asthma. No. 1 was a boy 8 years old, subject to fortnightly returns of three days duration, and I gave him fifteen drops fluid extract in an equal quantity of syr. ipecac, repeated every three hours. The first dose materially relieved him, and after taking the third dose he has been free from his asthma ever since, now full seven weeks.

No. 2, a man æt. 61, has had severe asthma for the last twenty-five years ; gave him thirty drops fluid extract with same quantity syr. ipecac, and ten grains bromide of ammonium in water ℥ ss, repeated every four hours ; this was at 8 o'clock at night. At 9 the next morning he presented himself free from his asthma, and says he has tried almost everything, but the above was the only thing that had such rapid effect.

No. 3 was a case of nervous asthma, in which I gave the man twenty-five drops of fluid extract in water, to be repeated every three hours, if necessary. He only took one dose which cut short the attack for the time, and when I have a full supply he is going to give the grindelia a fair trial te see if it will cure him."

---

**Treatment of Constitutional Syphilis by Sulphate of Copper.**

Drs. Martin and Oberlin (Medical Record) gave a brief report upon this subject at a late meeting of the Paris Academy of Medicine. The authors treated fifty patients, who showed various manifestations of syphilis, by the copper sulphate. The results were quite satisfactory, the fifty patients all being cured. A comparison of this method was made with the ordinary mercury methods, and it was found that the copper salt proved more efficacious and required less time for its beneficial action than did the mercury salts. The copper was also well borne by most patients. In only one case it produced initial vomiting, followed, however, by permanent tolerance of the drug. In a case of very grave syphilis, when mercury had proved useless, the administration of copper effected a rapid and complete cure. In a few patients the gums became affected, a greenish tint appearing at their free border. But this cupric gingivitis yielded more rapidly than the analogous mercurial affection ordinarily does. Actual sponginess of the gums was not observed. The salt was exhibited by the mouth in doses of one-sixteenth to one-sixth of a grain per day. An aqueous solution was employed. External application was also made by adding five drachms of the salt to a full bath.—*Gaz. Med. de Paris.*

**Cough.**

The following are the conclusions adopted in a report made by a committee of the New York County Medical Society :

1. Cerium oxalate may be given safely in doses of ten grains or more three times a day, for many days in succession.

2. The only symptom noted from such doses is a slight dryness of the mouth for the first few days.

3. It is probably more efficient when taken dry upon the tongue.

4. Its effects are not fully apparent until it has been taken two or three days and continued about the same length of time after its use is suspended.

5. For chronic cough it is best taken on an empty stomach early in the morning and at bed-time, with other doses during the day, if required, the initial dose for an adult being five grains.

6. It is in the majority of cases an efficient cough medicine, at least for a considerable time, and it is very valuable as an alternate with other drugs used for that purpose.

7. It does not disturb the stomach as do opiates and most other cough remedies; but on the contrary it tends to relieve nausea and to improve digestion.

8. The different preparations on the market are not of equal value, and when success is not obtained with one, another should be substituted.

---

**On Tonga.**

The results obtained from tonga by Drs. Ringer and Murrell fully coincide with mine. I have notes of cases of brain and kidney diseases in which tonga alone succeeded in removing pain. I shall, however, confine myself to reporting the effects upon the eye. Some months ago, when commencing experiments with tonga, I had the notion, the result of conversation with patients from the Fiji Islands, that the drug might have a specific effect upon nerves which are instrumental in pain.

Of the three preparations, tonga in a bag, the watery extract, and the alcoholic extract, I found the alcoholic extract alone reliable. When dropped into a healthy eye it seemed to increase the power of accommodation, to approach the nearest point of distinct vision, without affecting the size of the pupil (though in some cases, taken in large doses internally, it caused great dilation of both pupils). It acted beneficially in several cases of asthenopia. The sister in the eye wards gave it with great benefit to a man suffering from painful rheumatic iritis. Several patients with intolerance of light were rapidly relieved.

A most striking effect was obtained upon diminished tension of the eyeball. Two months ago a lady consulted me for intense pain in the right eyeball, with marked decrease of tension (T—2), intolerance of light, and watering, the pupil and cornea being clear with some conjuctival redness. The intense pain had deprived her of sleep for several nights. Some of the alcoholic extract of tonga was dropped into the eye at 2, 5, 7 and 9 P. M. The following day all intolerance of light had ceased, and she had passed a good night, free from pain. She stated that the drops caused no pain, but a sense of warmth, and that the pain in the eye subsided gradually ; their use was continued for several days. Remarkable was the rapidity with which the tension of the eyeball became normal and remained so.

All cases of neuralgia (supra and infra-orbital branches of the fifth nerve), with swelling of the temporal veins during the attack, were benefited. In these a teaspoonful of the extract in half a tumbler of water, and two or three more at an interval of half an hour until the pain subsided, were given.—*C. Bader, M. D., in Lancet.*

---

**Some of the Formulas of the Hartford, Conn, Hospital.**

Dr. Jas. P. Holt, in a communication to the *Medical and Surgical Reporter*, thus gives a brief account of the prevailing diseases and their treatment in the Hartford Hospital :

The most common diseases that occur are phthisis, intermittent fever, and alcoholism. They do not present any local peculiarities.

The treatment for intermittent fever consists in the administration of cinchonidia sulphate, in doses of from ten to fifteen grains, before the chill, and the continuation of five grains three times a day. This method usually controls the disease at once ; if not, the ten or fifteen grain doses are repeated before the time of the next chill, about one hour. During the chill stimulants are given moderately, and hot bottles applied. Constipation is present in a majority of cases, and the pil. cathartic, co. is given. This treatment generally suffices in nearly every case ; occasionally a case continues in spite of treatment. We have found that the hot air bath, given for an hour just previous to the time for the chill, controls the case where other treatment has failed. Other cases have been treated with three drop doses of Fowler's solution successfully, where cinchonidia sulphate has failed. The ordinary house diet is given.

The treatment for alcoholism consisted in the administration of beef tea, with capsicum, milk, eggs, hydrate chloral, potassium bromide, in doses of fifteen grains of the former and twenty grains of the latter once in two or three hours, as required. Under this treatment the cases progress very favorably.

The treatment of phthisis consists of the administration of cod-liver oil, whiskey, anodyne cough mixture, with some form of opium at night, if required ; special diet, consisting of anything which suits the patient's fancy, is allowed.

Among some of the house mixtures are :

BUCKLEY PILL.

℞
Sodæ bicarb.
Rhei rad. pulv., āā gr. ij.
Ipecac. pulv., gr. $\frac{1}{6}$.
M. Ft. pil. No. j.
Sig.—One t. i. d.

PILL SALUTIS.

℞
Pulv. aloes., gr. x.
Ext. hyoscyami, gr. x.
Ext. nucis vom., gr. ijss.
Ol. anisi., gtt. v.
M. Ft. pil. No. x.
Sig.—One at night.

COUGH MIXTURE, NO. 1.

℞

Ammon. carb., gr. viij.

Tr. scillæ., ʒ ij.

Syr. tolutan.,

Tr. opii camph., āā ʒ iv.

Decoc. senegæ, ℥ iij.

M. Sig.—Teaspoonful.

COUGH MIXTURE, NO. 2.

℞
Syr. pruns virg., ℥ j.
Sol. morphiæ Mag., ʒ j.
Acid. hydtocyl dil., gtt. viij.
Aquæ., ℥ iij.
M. Sig.--Teaspoonful.

---

**Medicated Pencils.**

In a paper read before the Paris Therapeutical Society, M. Legras recommends the following forms of local applications for the treatment of diseases of the eyes and face. They are intended as substitutes for the ordinary ointments officinal in the French Codex, but with slight modifications could readily be adapted to the formulas of the United States Pharmacopœia. They offer the advantage of greater cleanliness and convenience in their application. The different masses are to be rolled or cast into cylindrical pencils, and protected with waxed paper or tin foil, as the case may demand, with an outside covering of ordinary paper to provide against the heat of the hand :

YELLOW OXIDE OF MERCURY PENCILS.

Pure glycerine, $2\frac{1}{2}$ drachms.
Cacao butter, 5 "
Yellow oxide of mercury, 24 grains.

RED PRECIPITATE PENCILS.

Pure glycerine, $2\frac{1}{2}$ drachms.
Cacao butter, 5 "
Red precipitate, 24 grains.

OIL OF CADE PENCILS.

Pure glycerine, $2\frac{1}{2}$ drachms.
Cacao butter, 5 "
Pure oil of cade, 15 grains.

DEODORIZED IODOFORM PENCILS.

Pure glycerine, $2\frac{1}{2}$ drachms.
Cacao butter, 5 "
Iodoform, 1 ounce,
Oil of peppermint, 6 drops.

CORROSIVE SUBLIMATE PENCILS.

Pure glycerine, 3 drachms.
Cacao butter, 5 "
Corrosive sublimate, 15 grains.

BALSAM OF PERU PENCILS.

Pure glycerine, 75 grains.
Cacao butter, 7 drachms.
Balsam of Peru, 75 grains.
The resinous matter is to be separated.

NORWEGIAN TAR PENCILS.

Yellow vaseline, 75 grains.
Cacao butter, 5 drachms.
Norwegian tar, 75 grains.

OPHTHALMIC PENCILS.

Red precipitate, 45 grains.
Oxide of zinc, 45 "
Sugar of lead, 45 "
Burnt alum, 45 "
Corrosive sublimate, 7 grains.
Pure glycerine, $2\frac{1}{2}$ drachms.
Cacao butter, 5 "

—*Druggist's Circular.*

